# Fatigue-driven compliance increase and collagen unravelling in mechanically tested anterior cruciate ligament

**DOI:** 10.1038/s42003-023-04948-2

**Published:** 2023-05-26

**Authors:** Kevin H. Putera, Jinhee Kim, So Young Baek, Stephen H. Schlecht, Mélanie L. Beaulieu, Victoria Haritos, Ellen M. Arruda, James A. Ashton-Miller, Edward M. Wojtys, Mark M. Banaszak Holl

**Affiliations:** 1 Department of Chemical and Biological Engineering, Monash University, Clayton, VIC 3800 Australia; 2 Department of Chemistry, University of Michigan, Ann Arbor, MI 48109 USA; 3 Department of Mechanical Engineering, University of Michigan, Ann Arbor, MI 48109 USA; 4 Department of Orthopaedic Surgery, Indiana University School of Medicine, Indianapolis, IN 46202 USA; 5 Department of Biomedical Engineering, University of Michigan, Ann Arbor, MI 48109 USA; 6 Macromolecular Science and Engineering Program, University of Michigan, Ann Arbor, MI 48109 USA; 7 Department of Orthopaedic Surgery, University of Michigan, Ann Arbor, MI 48109 USA; 8 Department of Mechanical and Materials Engineering, University of Alabama at Birmingham, Birmingham, AL 35294 USA; 9 Department of Orthopaedic Surgery, Heersink School of Medicine, University of Alabama at Birmingham, Birmingham, AL 35294 USA

**Keywords:** Atomic force microscopy, Scanning probe microscopy

## Abstract

Approximately 300,000 anterior cruciate ligament (ACL) tears occur annually in the United States, half of which lead to the onset of knee osteoarthritis within 10 years of injury. Repetitive loading is known to result in fatigue damage of both ligament and tendon in the form of collagen unravelling, which can lead to structural failure. However, the relationship between tissue’s structural, compositional, and mechanical changes are poorly understood. Herein we show that repetitive submaximal loading of cadaver knees causes an increase in co-localised induction of collagen unravelling and tissue compliance, especially in regions of greater mineralisation at the ACL femoral enthesis. Upon 100 cycles of 4× bodyweight knee loading, the ACL exhibited greater unravelled collagen in highly mineralized regions across varying levels of stiffness domains as compared to unloaded controls. A decrease in the total area of the most rigid domain, and an increase in the total area of the most compliant domain was also found. The results highlight fatigue-driven changes in both protein structure and mechanics in the more mineralized regions of the ACL enthesis, a known site of clinical ACL failure. The results provide a starting point for designing studies to limit ligament overuse injury.

## Abstract (web summary)

A series of mechanical tests in human cadavers show the unravelling effect that mechanical loading has on collagen at the femoral enthesis of the anterior cruciate ligament

## Introduction

Non-contact ACL failure is responsible for about 75% of all ACL injuries^1,2^ with many of the injuries occurring at the femoral enthesis^3,4^. Recent studies on both ligament and tendon have demonstrated the susceptibility of collagenous tissue to experience material fatigue due to repetitive mechanical loading^5–10^. A key chemical marker of collagenous tissue fatigue has been identified as unravelling of the collagen molecule triple helix^6,7,10^, which results in structural change across the molecular, nano- and micro-length scales^6,7^ associated with the hierarchical structure of collagen tissue including triple helix molecules (1.5 × 300 nm), fibrils (~50–500 nm diameter), and fibres (~1–20 μm diameter)^11^. Although simulations provide insight into the mechanism responsible for the accumulation of unravelled collagen in collagenous tissue during repetitive fatigue loading^7^, it is not known how tissue mineralisation is associated with the morphology of the fatigue damage and how fatigue damage influences the mechanics of the ACL femoral enthesis. To address this knowledge gap, we employed atomic force microscopy infrared spectroscopy (AFM-IR) to simultaneously map the unravelled-intact collagen ratio, topography, and stiffness at the femoral enthesis for ACL explants from human cadaver knees. Data were collected from five pairs of ACLs from both male and female cadaveric knees between 25 and 47 years old. For each pair of knees, one knee underwent 100 cycles of mechanical fatigue loading to induce ACL strain simulating a jump pivot landing manoeuvre while the other contralateral knee served as an unloaded control. The 100 knee loading cycles were applied in a 3D direction (namely knee compression combined with simultaneous knee flexion and internal tibial rotation moments) at a magnitude demonstrated to biomechanically strain the ACL, but less than the load necessary to cause failure in a single cycle^6,12,13^. These applied loads are greater than those typically experienced in daily life and would typically only occur during strenuous athletic training manoeuvres involving a sudden stop, turn, or jump landing. The knee was inverted in the loading apparatus so that the drop weight applied an impulsive load to the distal end of the tibia just as a ground reaction force applies an impulsive reaction force to the bottom of the foot and distal tibia when landing a jump, stopping, or turning abruptly. The drop weight applied a loading rate such that the peak applied force and moments occurred after 70 ms, a value consistent with the published time course of the measured underfoot ground reaction force for a jump landing. The ACL explant was preserved in normal saline solution at 4 °C for <72 h, until cryo-embedding and sectioning into 20-µm thick sections. The sectioned samples were transferred to an adhesive tape via the Kawamoto method^14^ and stored at −20 °C until AFM-IR data collection.

## Results

### Fatigue-loaded ACL showed a higher unravelled/intact collagen ratio

**Fig. 1:**
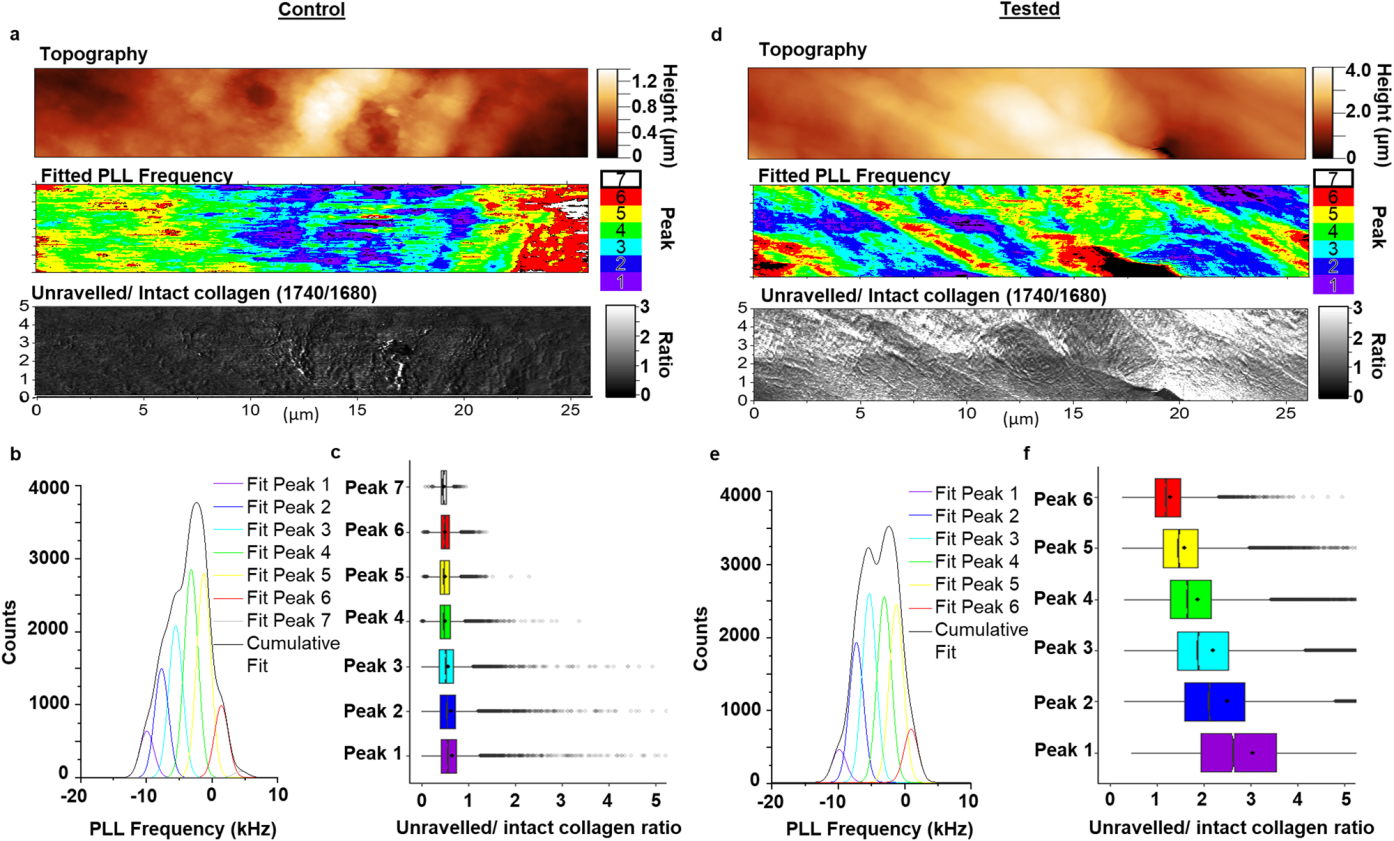
Representative data showing the relationship between collagen unravelling and stiffness domains between control and tested cadaver ACL (sample I, 42-year-old female). **a** Topography, fitted PLL frequency, and unravelled/intact collagen ratio map collected on a 5 × 26 µm ROI from the tidemark (left) into the ligament of the control ACL. **b** Distribution of PLL frequency map fitted with 7 peaks of equal width, which represent varying levels of domain compliance with Peak 1 being the most compliant. **c** Boxplot of unravelled/intact collagen ratio in each of the fitted peaks demonstrating the relationship of collagen unravelling with the different stiffness domains in control ACL. **d** Topography, fitted PLL frequency, and unravelled/intact collagen ratio map collected on a 5 × 26 µm ROI from the tidemark into the ligament of the tested ACL. **e** Distribution of PLL frequency map fitted with 6 peaks of equal width, which represent varying levels of domain compliance with Peak 1 being the most compliant. **f** Boxplot of unravelled/intact collagen ratio in each of the fitted peaks demonstrating an increasing collagen unravelling with increasing domain compliance in the ACL. Boxplot legend: solid dot = mean, solid line = median, whiskers = 5–95% of data, box = 1st–3rd interquartile range, Translucent dot = outliers, *n* = 2 human cadaver ACLs.

**Fig. 2:**
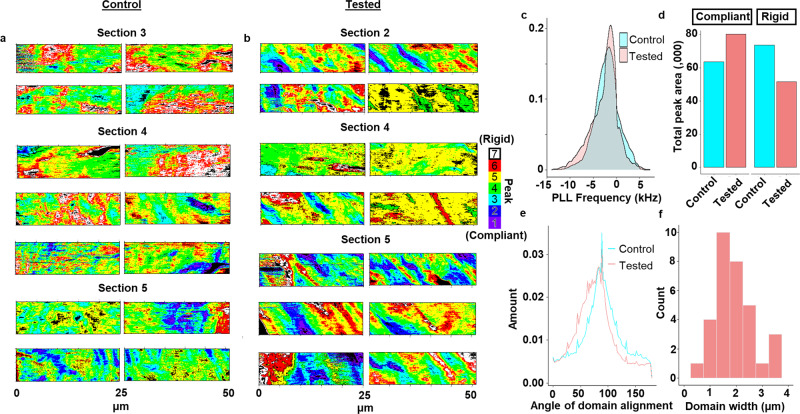
Change in morphology and compliance of stiffness domain in tested ACL. **a** Fitted PLL frequency maps (*n* = 14) along the tidemark (left) of the control ACL across three separate ACL sections of cadaver sample I. **b** Fitted PLL frequency maps (*n* = 14) along the tidemark of the tested ACL across three separate ACL sections of cadaver sample I. **c** Kernel density of all PLL frequency maps across all the ACL sections of cadaver sample I between control and tested ACL. The KS test indicates the change is statistically significant (*p* < 2.2 × 10^−16^). **d** Quantification of the change in total peak area in the more compliant range (Peak 1, 2, 3) and in the more rigid range (Peak 6, 7) across all the ACL sections of cadaver sample I between control and tested ACL. **e** Comparison of the overall angle of stiffness domain alignment across all fitted PLL frequency maps between control and tested ACL. **f** Width distribution of the stiffness domain for the fitted PLL frequency in the tested ACLs that demonstrated striation. *n* = 2 human cadaver ACLs.

**Fig. 3:**
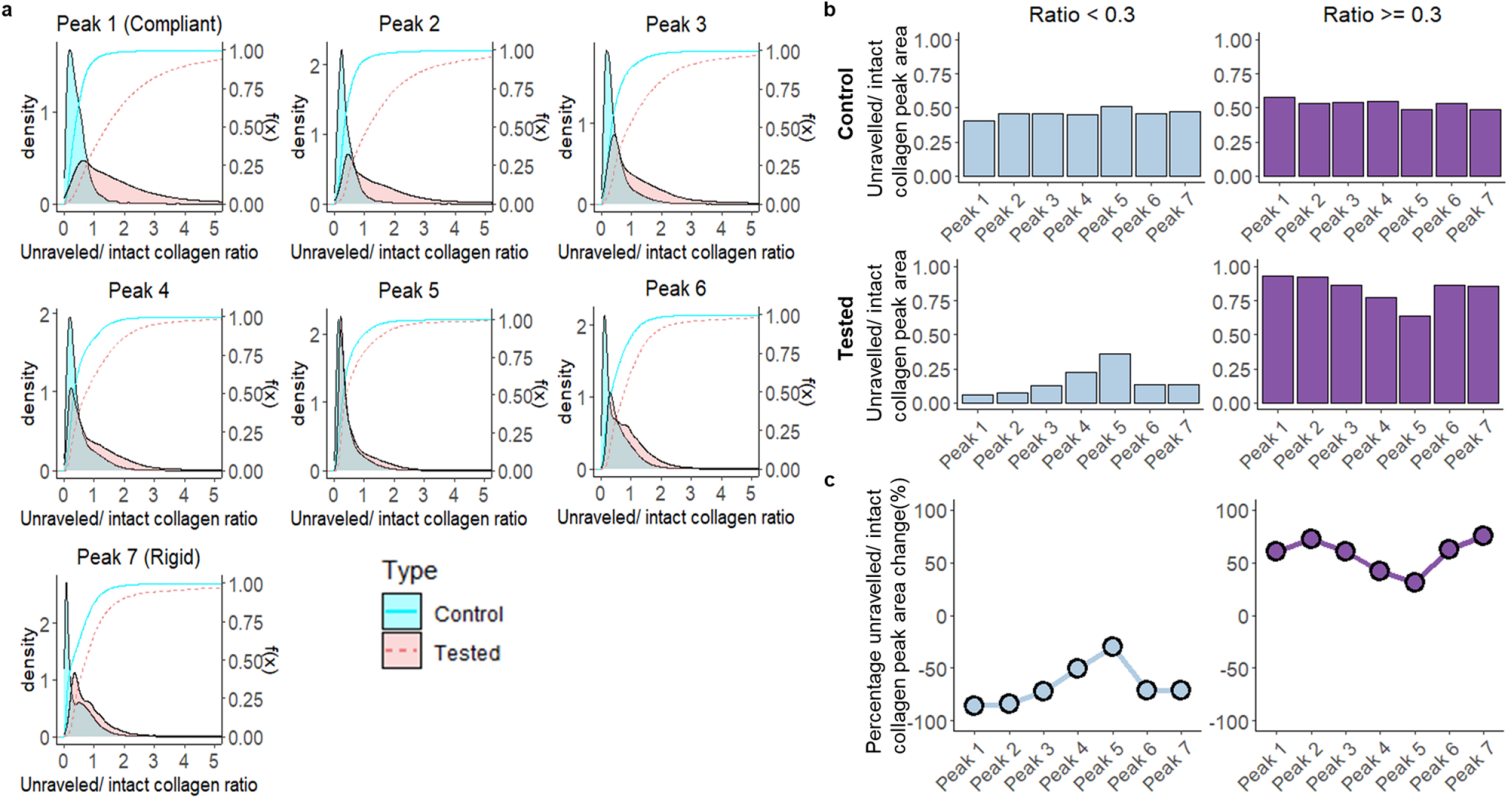
Fatigue testing resulted in greater amounts of unravelled collagen across all stiffness domains of cadaver sample I. **a** Comparison of the kernel density distribution of the unravelled/intact collagen ratio as a function of fitted peaks in the control and tested ACL (KS test; *p* < 2.2 × 10^−16^). **b** The area under the kernel density of the unravelled/intact collagen ratio (**a**) segregated by ratio segments: ratio <0.3 and ratio ≥0.3 in control and tested ACL. This is shown as a proportion of the ratio segment in each of the fitted peaks between control and tested ACL across all the sections of cadaver sample I. **c** Percentage differences were calculated based on the change in area of the unravelled and intact collagen peaks as a result of fatigue testing. *n* = 2 human cadaver ACLs.

**Fig. 4:**
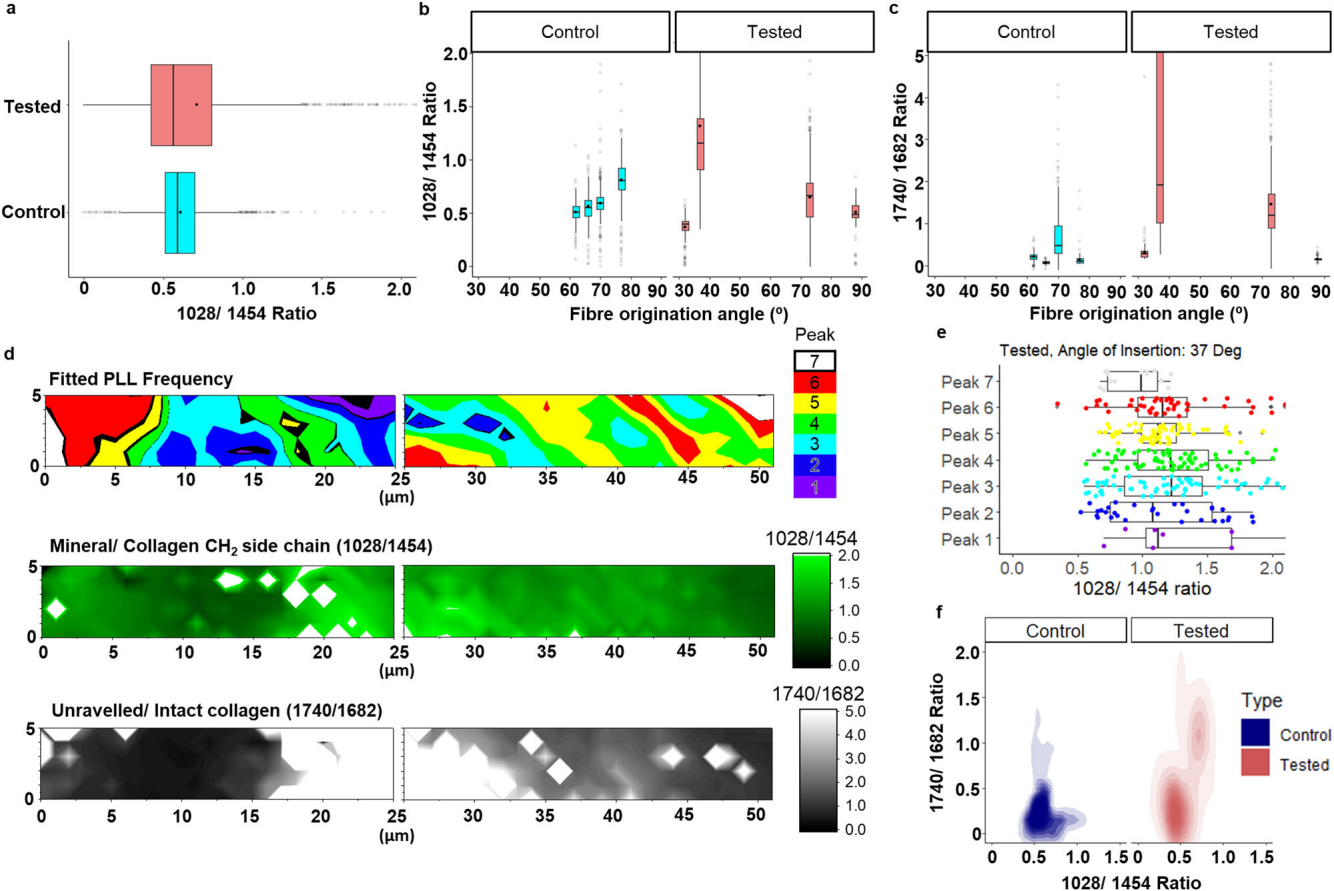
Localisation of fatigue-induced collagen unravelling in regions with higher mineralisation in cadaver sample I. **a** Comparison of the boxplot of the mineral/collagen CH_2_ side chain (1028/1454) ratio between the control and tested ACL. **b** Boxplot of the mineral/collagen side chain ratio across various locations represented by the local bulk fibre angle along the tidemark (left) between control and tested. **c** Boxplot of the unravelled/intact collagen ratio across various locations represented by the local fibre origination angle along the tidemark between control and tested. **d** Fitted PLL Frequency, mineral/collagen CH_2_ side chain ratio and unravelled/intact collagen ratio map collected up to 50 µm from the tidemark in the ligament of the tested ACL. **e** Boxplot of mineral/collagen side chain ratio in each of the fitted peaks demonstrating an increasing mineralisation with increasing domain compliance in the tested ACL. **f** Comparison of overall 2D density plot of the mineral/collagen CH_2_ side chain ratio against unravelled/intact collagen ratio between the control and tested ACL showing overall increase in collagen unravelling with increasing mineralisation post testing. Boxplot legend: solid dot = mean, solid line = median, whiskers = 5–95% of data, box = 1st–3rd interquartile range, Translucent dot = outliers, *n* = 2 human cadaver ACLs.

In a previous study^6^, the presence of a 1740 cm^−1^ spectroscopic band was assigned to unravelled triple helix collagen molecules^7–10^ was identified in ACL sections taken from fatigue-tested human cadaver knees as well as in samples from patients who had recently suffered an ACL injury. However, no information on the morphology of the unravelled collagen and the relation to mechanical changes at the femoral enthesis was obtained. To elucidate the location of the unravelled collagen and its relationship to mechanical changes at the femoral enthesis, 10 mm trephined ACL explants from the control and tested knees were removed using a guided outside-in procedure with the knee flexed ~90°^6,12,13^. We first stained ACL sections with Toluidine Blue (Supplementary Fig. 1) to visually distinguish the bone and ligament and identify the tidemark where the bone and ligament meet. Anatomical orientation of the explants was maintained throughout and was consistent for both control and loaded samples, which consisted of both AM and PL bundles and showed variation in acuteness of fibre entry. We next performed single IR wavenumber spatial mapping employing the characteristic unravelling signature (1740 cm^−1^) up to 50 µm into the ligament from the tidemark (Fig. 1a, d). Two sets of data were collected: cadaver sample I was collected with our most detailed imaging protocol and cadaver samples II, III, IV and V with less detail and data points (Supplementary Table 1). Hyperspectral imaging including measurement of relative stiffness was obtained for sample I over a series of contiguous 5 × 50 µm regions at the femoral enthesis using ten regions of interest (ROIs) and generating 3180 unique spectra/pixels over the 2500 μm^2^ area imaged. The other four 5 × 50 µm regions were acquired using high-resolution (~50 nm) single-wavenumber chemical mapping (1680 and 1740 cm^−1^), which also yielded relative stiffness mapping. These sample I data are highlighted in Figs. 1–4 and Supplementary Figs. 1 and 2. To extend the observations and test the conclusions reached from sample I and to reduce total imaging required for the study by roughly an order of magnitude, another four paired human cadaver knees were examined using one to two 5 × 5 µm ROIs at a distance of 40 and 80 µm from the tidemark for each tissue section resulting in a total of 268 unique spectra/pixels over the 750 μm^2^ area imaged. The sample II–V data are highlighted in Supplementary Figs. 3, 4 and 7. Supplementary Figs. 5 and 6 employ data from samples I–V.

Consistent with our previous study^6^, the fatigue-tested ACL exhibited a higher unravelled/intact collagen ratio (1740/1680) at the femoral enthesis as compared to the contralateral control in all five cadaver samples (Supplementary Figs. 2c, 3c and 4). The control ACL did exhibit collagen unravelling, suggesting that there is a physiological level resulting from previous loading activity and/or natural collagen turnover (Supplementary Fig. 2a).

### Quantitative comparison of PLL frequency data

To determine the effects of compositional change on the tissue mechanics, we collected stiffness information simultaneously with chemical information using the Phase-locked loop (PLL) frequency channel (Fig. 1a, d). The PLL frequency measures the contact resonance frequency of the probe-sample interaction, with high frequency indicating a stiffer surface and a lower frequency indicating a more compliant surface^15,16^.

We then processed the PLL frequency data by fitting up to seven peaks on the PLL frequency map distribution and scaling them to Peak number 5, which was defined as 0 kHz (Fig. 1b, e). The peak fitting allows quantitative analysis of stiffness domains and sample-to-sample comparison, with Peak 1 being the most compliant domain and Peak 7 being the most rigid domain. Furthermore, we quantified the unravelled/intact collagen ratio in each of the fitted peak domains and compared between the amounts of collagen unravelling in the fitted peak domains (Fig. 1c, f).

A representative ROI from the tested ACL of cadaver sample I (Fig. 1f) showed higher collagen unravelling across all the stiffness domains as compared to the control (Fig. 1c). Furthermore, the greatest amount of collagen unravelling was found to be at the most compliant domain for the tested case (Fig. 1f), whereas there was no observed difference in collagen unravelling between all the stiffness domains for the control case (Fig. 1c).

A broader look across all the ROIs for three separate ACL sections for the control and tested ACL of cadaver sample I is consistent with the representative data (Fig. 1c, f), which showed the tested ACL having significantly higher collagen unravelling across all the stiffness domains, with the greatest collagen unravelling at the most compliant peak (Supplementary Figs. 2a, 2b and 3c and Fig. 3a, *p* < 2.2 × 10^−16^). When we compare the overall kernel density distribution of the PLL frequency across all the ROIs, we observed significantly lower density of the high frequency (>0 kHz) and higher density of the low-frequency domains (<−5 kHz) as compared to the control dataset (*p* < 2.2 × 10^−16^) (Fig. 2c). Quantifying these differences showed that the rigid stiffness domain areas decreased by ~30%, whereas there was a ~30% increase in the compliant domain across all the ROIs of cadaver sample I (Fig. 2d). These results indicate that cyclic loading causes an increase in collagen unravelling, a reduction in nano-scale rigid domains, and an increase in nano-scale compliant domains at the ACL enthesis. The concomitant loss of tissue stiffness and collagen damage suggests a possible nanomechanical path for how bulk mechanical failures of the ACL can eventuate based on the multi-scalar impact of fatigue^6^. Overall analysis on the sum of the data from cadaver samples II, III, IV and V were consistent with that from cadaver sample I in showing a significantly greater amount of unravelled collagen and a reduction in the most rigid domain in the mechanically tested ACL (Supplementary Fig. 3a–c; *p* < 2.2 × 10^−16^).

### Fatigue increases compliant domain area and changes stiffness domain morphology

In order to explore the relationship of the applied mechanical load to changes in the bone-ligament connection, we measured the angle of the stiffness domain alignment parallel to the tidemark of the tested ACL. We observed a shift in distribution toward a more acute angle (75°), whereas the control ACL showed a distribution centred towards normal to the tidemark (89°) (Fig. 2e). The difference in the angle of domain alignment is statistically significant based on a two-sample *t*-test (*p* < 9.4 × 10^−5^). A previous study^17^ had shown that the collagen fibres in the normal ACL originate from the femur at a less acute angle than observed in the mechanically tested sample, thereby reducing the strain concentration at the femoral enthesis^18^, which is what is observed in this study for the control sample. The width of the aligned stiffness domains is around the size scale of a collagen fibre (1.5 µm) (Fig. 2b, f), consistent with previous reports collagen fibre deformation at the attachment site under load^19^. This observation suggests that the observed variation in stiffness caused by collagen unravelling is manifesting at the level of fibre property changes. Collectively, these results indicate that when the ACL undergoes fatigue, the micro-scale structure forms striations with an increase in the area of compliant domains in response to fatigue loading.

### Highest unravelled/intact collagen ratio occurs in the most compliant domain

A key aspect of this study was to identify how tissue structural and compositional changes were related to changes in tissue mechanics as a function of material fatigue testing. The peak fitting of PLL frequency data allows a holistic comparison across all sample I sections (Fig. 2a, b). Earlier, it was noted that the mechanically tested ACL had a greater unravelled/intact collagen ratio across all the stiffness domains in sample I sections (Supplementary Fig. 3c). We then investigated in detail the difference in the overall kernel density distribution of the unravelled/intact collagen ratio across all ROIs collected between the control and tested ACL as a function of stiffness domain (Fig. 3a). Across all the stiffness domains in cadaver sample I, the kernel density distribution from the tested ACL showed greater density in the areas with higher unravelled/intact collagen ratio compared to the control with differences increasing as the domains become more compliant. This observation is consistent with our previous analysis (Supplementary Fig. 3c) and the overall analysis from samples II–V (Supplementary Fig. 4).

To quantify these differences, we segregated the data into unravelled/intact collagen ratio <0.3, which represents regions with a physiological level of collagen unravelling, and ≥0.3 which represents damaged regions containing high levels of collagen unravelling. We established 0.3 as a cut-off ratio based on our collected IR spectra, which show that unravelled/intact collagen ratio (1740/1680) <0.3 in both control and tested groups is consistent with a typical shoulder seen in IR spectra collected from healthy collagenous tissue (Supplementary Fig. 5). It is only when the ratio is greater than 0.3 that a distinct peak is observed at 1740 cm^−1^, thereby deviating from what is considered normal, healthy collagenous tissue spectra. We calculated the relative amounts of physiological levels of unravelled collagen and high levels of unravelled collagen within each of the stiffness domains (Fig. 3b) and quantified the difference between control and tested ACL (Fig. 3c). The results demonstrate that all the stiffness domains had lower amounts of a physiological level of unravelled collagen (<0.3) and higher levels of unravelled collagen (≥0.3) due to fatigue loading (Fig. 3c). Furthermore, the most compliant and rigid domains exhibited the greatest percentage change in collagen unravelling as compared to control. This suggests that the rigid domains play a role in dissipating energy during fatigue loading through the accumulation of fatigue-induced collagen unravelling.

### Collagen preferentially unravels in highly mineralised regions

So far, we have shown that overall collagen unravelling is greater in the tested ACL, which suggests a mechanism for the increase in overall compliance compared to control. However, the amount of fatigue-induced collagen unravelling varies along the tidemark and from section to section where the ligament originates from the femur (Supplementary Fig. 2a, b). This observation is consistent with previous modelling-based studies showing that shear strain is distributed heterogeneously across the ACL, with the tidemark profile and origination angle influencing the level of shear strain experienced in the ACL^18,20^. The observations are also consistent with an elastography imaging study that indicated the presence of both compressive and tensile strains that are heterogeneously distributed across the enthesis^21^. However, it is unclear whether tissue mineralisation may influence the distribution of fatigue along the tidemark. We collected hyperspectral imaging data at the same locations along the tidemark across a subset of our data from cadaver sample I (Supplementary Fig. 2a, b), where each pixel contains a full IR spectrum in the 1800–800 cm^−1^ range. This allows us to assess the change in mineralisation through the hydroxyapatite mineral phosphate/collagen CH_2_ side chain (1028/1454) ratio along with the unravelled/intact collagen ratio calculated from each IR spectra. The overall mineral content is not statistically different (*p* = 0.86) between the control and tested ACL across all the cadaver samples (Fig. 4a and Supplementary Fig. 6a, b). We also collected IR spectra from cadaver samples II, III, IV and V, and performed an overall analysis to evaluate the change in mineral ratio with unravelled collagen ratio (Supplementary Fig. 7).

Here we provide experimental evidence of fatigue-induced collagen unravelling occurring more preferentially in entheseal sites with greater mineralisation, which is believed to be a mechanism to dissipate energy during loading^22,23^. Further analysis of cadaver sample I showed variations in mineral ratio (Fig. 4b) and unravelled collagen ratio (Fig. 4c) with location and local fibre origination angle along the tidemark. Data for the tested specimen at 37° fibre origination angle are illustrated in Fig. 4d. However, tested ACL locations with higher mineral content had a significantly more acute local fibre origination angle (*t*-test; *p* < 9.4 × 10^−5^) and more unravelled collagen. Given previous work showing that mechanical loading induces a more acute angle at the insertion point^19^, this suggests that the heterogeneous nature of mineralisation in the enthesis region generates a distribution of loads as fibres insert into the bone matrix.

Overall analysis of mineral matrix ratio and unravelled collagen ratio across all locations and tissue sections in cadaver sample I showed clusters of high mineralisation (ratio >0.7) with high collagen unravelling (ratio >0.3) in the tested ACL as compared to control (Fig. 4f). Results from k-means clustering of the tested dataset agrees with our observation in producing 2 distinct clusters: cluster 1 (centroid: 0.5, 0.3) and cluster 2 (centroid: 0.7, 1.3), where cluster 2 contains a region with higher mineralisation and higher collagen unravelling compared to cluster 1. This suggests that higher unravelled collagen ratio is most likely to be found in regions with higher mineral ratio in the tested ACL. Similarly, analysis of cadaver samples II, III, IV and V also show an increase in collagen unravelling with increasing mineralisation post testing (Supplementary Fig. 7). Interestingly, the same region of greater collagen unravelling (Fig. 4c; origination angle of 37°) and mineralisation (Fig. 4b) are co-localised with the most compliant stiffness domains in the tested ACL (Fig. 4d, e).

Previous modelling studies have shown that the presence of mineralised collagen fibrils increases rigid mechanical properties as opposed to collagen fibril alone^22^. Furthermore, it has been shown that the stress is four times more concentrated in mineral-rich regions as compared to collagen-rich regions, whereas the collagen-rich region undergoes more strain and deformation than the mineral-rich region. Qin et al. have shown through modelling of tropocollagen and hydroxyapatite together that collagen triple helix unravelling occurs at the collagen-mineral interface in response to mechanical loading^22^. Nair et al. suggested that the interface of the mineral-rich region and the collagen-rich region is essential as it allows energy associated with local stress concentrated in the mineral-rich domain during loading to be transferred to the nearby collagen-rich region, where it dissipates the energy through fibril deformation and collagen triple helix unravelling^23^. Our results are consistent with these studies and in a physiologically relevant intact ACL in the human knee support the hypothesis that fatigue-induced collagen unravelling at the more highly mineralised entheseal site generates an increase in compliance and a reduction in rigidity.

## Discussion

Work by Weiss et al. has demonstrated molecular unravelling of collagen by applying controlled mechanical loads to isolated rat tail tendon fascicles and flexor digitorum longus tendons^7,8,24^. These studies have employed fluorescently labelled collagen hybridising peptide (CHP) to bind to the unravelled regions, and by comparing to trypsinization, have been able to quantify the amount of unravelling present prior to mechanical loading (~0.25% of total collagen) and after mechanical loading, which is up to 2.5% under conditions reported. In both the control and mechanically loaded samples reported here, substantial regions are observed containing spectra with little to no intensity in the 1740 cm^−1^ region, consistent with large regions of intact collagen molecules as illustrated in Fig. 1a and Supplementary Fig. 2. However, Fig. 1d exhibits substantial regions of 1740 cm^−1^ intensity that are sufficient to raise the 1740/1680 ratio above 1 for much of the section, implying at least a local collagen unravelling density that may substantially exceed 2.5%. In our previous study, we demonstrated that an increase in 1740/1680 ratio as measured by AFM-IR was related to the increase in fluorescent CHP binding^6^, so it is interesting to consider the differences in the studies that may be leading to the apparently substantial difference in estimated collagen unravelling.

The series of studies by Weiss et al. and those described here differ substantially in terms of the nature of mechanical load applied, the biomaterials studied, and the detection mechanism for the collagen unravelling. Weiss et al. have employed uniaxial, monotonic single or cyclic loadings of tendon fasicles^7,24^ and flexor digitorum longus tendons^9^. By way of contrast, our studies reported herein and previously^6,25^ employ dynamic, multiaxial loading of the complete ACL organ connected via the partially mineralised enthesis region to both femur and tibia in an intact knee. The substantially different loading protocols may have led to a higher level of collagen unravelling in this ACL study. In addition, the greatest extent of unravelling for the ACL is noted in the mineralised enthesis region at the femoral connection, which is an aspect of this study fully absent in the isolated tendon work. By way of contrast, the region where the tendon interacts with the clamp connectors, which may be under the greatest strain, was trimmed away in these studies of tendon and not included in the assessment of total collagen unravelling. Thus, the ACL and tendon studies to date are focusing on substantially different tissue regions and mechanical loading protocols.

Finally, the AFM-IR experiment itself, as performed in this study, has an *x*–*y* resolution of ~50 nm and an expected *z*-resolution of a few hundred nm. Thus, spectra measurements will typically be obtained from regions at the level of the single collagen fibril level in *x*–*y* and at level of 1–3 fibrils in *z*. The data reported herein, with 1740/1680 ratios indicating up to 50–75% and sometimes greater in particular spectra, suggest substantial molecular collagen unravelling is present in individual collagen fibrils (these percentages can only be considered rough estimates as the relative oscillator strength and excitation cross-section contributions to the 1740 and 1680 cm^−1^ bands are unknown). Other spectra show little evidence of collagen unravelling, in the fibril-level assessment region. Thus, at least for the ACL, our study suggests that dynamic, multiaxial mechanical loads are inhomogeneously transmitted at the level of the collagen fibrils making up the ACL, causing molecular collagen unravelling that appears to self-segregate at the fibril level.

### Limitations and implications

The results demonstrate the use of AFM-IR to perform spatial spectroscopic analysis of fatigue at the femoral ACL enthesis induced by repetitive loading and the ability to relate this to the changes in the sectioned tissue nanomechanics. Through AFM-IR analysis, we showed that the fatigue-loaded ACL had a higher level of unravelled collagen localised at the most compliant and highly mineralised domains, post testing. These results agree well with the nanomechanical analysis where there was an increase in the total area of the compliant domain and a decrease in the total area of the rigid domain in the tested ACL. Therefore, our results suggest that fatigue-induced collagen unravelling increasingly happened at the most mineralised entheseal sites along the tidemark and drives a change in tissue mechanics leading to reductions in rigid domains and an increase in compliant domains. We note the limitation that these mechanical conclusions are drawn from transverse measurements on thin ligament sections and are inferred from relative change in the contact resonance frequency of the AFM cantilever. In summary, the study capitalises on the spectroscopic and mechanical capability of AFM-IR to further understand fatigue with nano-scale detail at the ACL enthesis.

We acknowledge that the contralateral control of the study is not perfect when comparing between the tested and control with different knees experiencing different loadings across the lifespan of the person. However, studies examining the progression of collagen unravelling in both ligament and tendon undergoing fatigue loading showed increasing progression of unravelled collagen with an increasing number of loading cycles^7,8,10,25^. This is consistent with our findings where we observed a greater amount of unravelled collagen in the fatigued ACL compared to the contralateral control.

Despite these limitations, our study highlights the morphology of fatigue damage in the ACL enthesis, which was greater in the mineralised region and affected the tissue mechanics at the nano-scale level. These findings provide a clearer view of the mechanism associated with ACL fatigue failure by relating the compositional changes associated with fatigue to the tissue mechanics, including elucidating a compositional difference that influences the localisation of fatigue.

We wish to highlight two possible avenues for future research to build on this study: (I) expand upon the present results using a larger sample of tested cadaver ACLs tested and taking advantage of the rapid instrumental improvements that should improve imaging sensitivity, relation to mechanical properties, and total experimental time required; (II) perform the present analyses on the proximal end of torn ACLs removed, using the same trephine drilling approach, from patients undergoing ACL reconstruction surgery, as described in Chen et al.^6^. The combination of these two approaches would help corroborate the present results and extend them to patients who have undergone ACL failure.

## Methods

### ACL sample preparation and testing

Pairs of fully de-identified adult donor knees were harvested at the University of Michigan Medical School and Gift of Life Michigan within 48 h of death after receiving an exempt determination for the experimental protocol from the University of Michigan Institutional Review Board. One knee was then randomly selected from each pair of knees and subjected to mechanical fatigue testing, while the contralateral knee was stored as an untested control. The fatigue testing was performed using a custom mechanical apparatus (Supplementary Fig. 8) that simulated 3D impulsive knee loads known to cause ACL strain during a jump pivot landing of 3–4 times bodyweight repeated for 100 loading cycles^5,6^. Next, the ACLs were explanted from both the control and tested cadaver knees while ensuring the connection between the femur and ligament was intact. The ACL explants were then embedded in a water-soluble embedding medium before cryosectioning at −25 °C using tungsten carbide blade to produce 20-μm-thick ACL sections. These ACL sections were transferred to polyvinylidene adhesive tape via the Kawamoto method^14,26^ and stored at −20 °C prior to AFM-IR analysis.

### Toluidine blue staining and brightfield imaging

The ACL section to be imaged was removed from −20 °C and washed by running deionised water across the surface for approximately 30 s to remove the embedding medium. Next, three drops of toluidine blue (concentration 0.5 wt %) were administered on the ACL and allowed to stain the tissue for approximately 30 s. The excess dye was then rinsed off using deionised water. Hydration of the tissue was maintained using a moisture chamber.

Brightfield imaging at ×5 and ×10 magnification was performed with a Nikon Eclipse Ni-U upright microscope (Nikon Instruments, Inc., Melville, NY, USA). A region with minimal tissue tear and the continuous bone-to-ligament interface was then identified for AFM-IR analysis. The tissue sitting on the polyvinylidene adhesive was then transferred to an AFM puck using a double-sided adhesive and allowed to rest at room temperature in a moisture chamber for 45 min to allow thermal equilibrium to be established.

### AFM-IR data collection

The AFM-IR utilised in this study was a Bruker NanoIR3 system (Bruker, Santa Barbara, CA, USA) equipped with a contact-mode gold-coated silicon nitride probe (PR-EX-nIR2-10) with a spring constant of 0.07–0.4 N/m and resonant frequency of 13 ± 4 kHz (Bruker), a Quantum Cascade Laser and a photo-detector capable of spectroscopic detection ranges of 790–1890 cm^−1^. AFM-IR was employed for the simultaneous collection of topography, single-wavenumber IR intensity maps, and PLL frequency map (relative stiffness). For ACL sample I, two to three 5 × 50 μm ROI were obtained by using adjacent 5 × 25 and 5 × 26 μm windows with 1 μm overlap for image stitching. For samples II–V, one 5 × 5 μm ROI or two 5 × 5 μm ROIs separated by 40 μm from each other moving away tidemark were employed. Toluidine staining (Supplementary Fig. 1) was used to indicate tidemark location. This was found to co-locate with a ~1000 nm offset in the topography image caused by microtome sectioning of the unfixed tissues making this a convenient reference point to the tidemark during AFM imaging. Simultaneous mechanical data collection was performed by implementing a PLL frequency feedback loop on the probe-sample contact resonance frequency with I gain = 6, P gain = 10 and a range between 150 and 190 kHz. Two single-wavenumber IR intensity maps associated with Type 1 collagen’s Amide I peak of intact triple helix (1680 cm^−1^) and unravelled helix (1740 cm^−1^) were collected using 3.37% laser power and 2% duty cycle. Hyperspectral data were collected immediately after the map collection on a number of locations at 1 µm spatial resolution. All the AFM-IR-produced maps and hyperspectral data were then exported for data analysis.

### Local fibre attachment angle measurement

The fibre attachment angle, defined as the angle between a line of best fit to the entheseal surface (tidemark) and an average line of best fit to the direction of the non-calcified fibrocartilage fibres, was measured in each of the regions where AFM-IR data was collected using custom Matlab code from the brightfield images of the toluidine blue stained ACL. The entheseal surface line of best fit was obtained by digitising the tidemark, interpolating the digitised points with a cubic spline, and fitting a first-order (linear) polynomial curve to the interpolated data. Similarly, the average fibre line of best fit was obtained by digitising the fibres from the tidemark to a distance of about 300–400 μm at five locations approximately equidistant to each other. At each location, the digitised points were interpolated with a cubic spline. Finally, the interpolated data were averaged along one dimension and fit with a first-order (linear) polynomial curve to get the average fibre line of best fit.

### PLL frequency peak fitting

The PLL frequency data measured the contact resonance frequency of the probe-sample interaction, with a feedback loop centring the phase shift to 0°. As a result, the PLL frequency represents the relative nano-stiffness measurement across the surface with a higher frequency indicating a stiffer domain, while a lower frequency indicating a more compliant domain. However, the absolute frequency values are affected by variation in probe spring constant (15–50 kHz) and normal force during probe engagement (2–6 kHz), which pose a challenge in comparing absolute frequency values directly. To address this, a method of allowing quantitative comparison between frequency data collected with different cantilever probe and samples was devised, which aligns the absolute frequency values through fitting the PLL frequency data distribution with Gaussian peaks of equal full width half max (FWHM). Each fitted peak represents a physically relevant domain with varying relative stiffness that can be transformed into a fitted frequency map to visualise the difference in stiffness domains as a function of peak fitted. When comparing the fitted frequency map with the raw frequency map collected directly from the instrument, the fitted frequency map is able to reproduce the stiffness domains from the raw frequency map. In addition, the peak fitting allows viewing of stiffness domains that cannot be viewed under consistent colour scales, quantitative analysis of stiffness domains (Supplementary Figs. 9 and 10), and comparison of frequency data collected with different cantilever probe and samples.

Individual PLL frequency distribution (Supplementary Fig. 10a) was generated from each of the ROIs (for example, see Supplementary Fig. 10c–e) and the associated raw PLL frequency maps (Supplementary Fig. 10e) collected on the ACL samples from the instrument. The PLL frequency distribution was then deconvoluted by fitting up to seven Gaussian peaks with equal FWHM. Each peak represents a physically relevant domain with distinct relative stiffness and is allowed to shift in peak position and height to fit the shape of the frequency distribution. Seven peaks with 2.5 FWHM were observed to be the minimum number of peaks required to best fit the dataset consisting of 28 PLL frequency distributions from 6 different ACL sections while able to reproduce the raw PLL frequency map.

The leading higher frequency edge of each distribution was fitted with peak number 5, which accounts for the most dominant peak of the PLL frequency distribution. Peaks are then added to the rest of the lower and higher frequency side of the distribution depending on the width and shape (Supplementary Fig. 10a).

### Creation of fitted PLL frequency maps

Using the statistics generated from the peak fitting (Supplementary Fig. 10b), the range of frequency values for each peak was calculated using the peak centre position ±0.5 FWHM, such that any absolute frequency values within the range of frequency values calculated were classified to be of that peak or stiffness domain. Generating the fitted PLL frequency map (Supplementary Fig. 10f) was done by segregating the absolute frequency values within each PLL frequency map according to the range of frequency values calculated for each individual fitted peak. Overlaps in the range of values between one peak number to the next peak were addressed by assigning the lower range value of the overlapped peak with the upper range value from the previous peak.

Quantitative analysis was then performed based on the classification of the PLL frequency and the corresponding pixel from the unravelled/intact collagen ratio maps into each of the fitted peak segments, where peak number 1 is the most compliant while peak number 7 is the most rigid.

### Statistics and reproducibility

All acquired maps were exported from the instrument and flattened into table format in which a single row of observations contains information such as the topography, PLL frequency, and IR ratio values. All statistics and data analysis were then carried out in RStudio based on R version 3.6.1 and Python. Normality was assessed by Shapiro–Wilk test. If normality was not met, the non-parametric Kolmogorov–Smirnov (KS) test or Mann–Whitney *U* test was performed. If normality was confirmed, an *F*-test was performed to test the homogeneity in variances. A two-sample *t*-test was then performed with equal variance. *P* values were considered significant if *p* < 0.05. Cluster analysis using K-means clustering was also performed. Graphical representation was carried out using ggplot2^27^. Plane fitting of the topography map and IR ratio map calculation were done using Bruker’s custom software, Analysis Studio version 3.15. Fitted PLL frequency and IR ratio map were plotted using the colour contour plot in OriginPro 2018, while topography maps were plotted using Gwyddion. Kernel density distributions were plotted from the overall dataset across all locations where data were collected. Bandwidths used for kernel density were based on Silverman’s ‘rule of thumb’^28^. Area under the curve was calculated using an adaptive quadrature of function approximation of the data over a finite interval. Angle of stiffness domain measurement was performed using ImageJ’s Directionality plugin through the Local Gradient orientation method^29,30^. The directionality histogram for each map was then exported and all the histograms averaged as control or tested. The exported hyperspectral data was analysed via Python. Wavenumbers of interest (1028, 1338, 1454, 1682, 1740) were selected to make ratios 1028/1338, 1028/1454 and 1740/1682, which were shaped into either a 6 × 26 or 6 × 27 matrix to match the hyperspectral spectral imaging dimension.

### Reporting summary

Further information on research design is available in the Nature Portfolio Reporting Summary linked to this article.

## Supplementary information

Supplementary Information

Reporting Summary

## Data Availability

The data that support the findings of this study are available from Figshare 10.26180/22770254.

## Code availability

The custom code used to generate the findings of this study is available from Figshare 10.26180/22771094.
