# Supplementary material for: Fatigue-driven compliance increase and collagen unravelling in mechanically tested anterior cruciate ligament
Source: Commun Biol. 2023 May 26;6:564. doi: 10.1038/s42003-023-04948-2 (PMC10219950; doi:10.1038/s42003-023-04948-2)
Supplement: Supplementary file 1 — Supplementary Information [file 42003_2023_4948_MOESM1_ESM.pdf]

Supplementary Information

Fatigue-driven Compliance Increase and Collagen Unraveling in Mechanically Tested Anterior Cruciate Ligament

Kevin H. Putera,[1] Jinhee Kim,[1,2] So Young Baek,[3] Stephen H. Schlecht,[4] Mélanie L. Beaulieu,[5] Victoria Haritos,[1] Ellen M. Arruda,[3,5,6] James A. Ashton-Miller,[3,5] Edward M. Wojtys,[7] Mark M. Banaszak Holl[1,8,9*]

[1]Department of Chemical and Biological Engineering, Monash University, Clayton, Victoria, 3800, Australia
[2]Department of Chemistry, University of Michigan, Ann Arbor, Michigan, 48109, United States
[3]Department of Mechanical Engineering, University of Michigan, Ann Arbor, Michigan, 48109, United States.
[4]Department of Orthopaedic Surgery, Indiana University School of Medicine, Indianapolis, Indiana, 46202, United States
[5]Department of Biomedical Engineering, University of Michigan, Ann Arbor, Michigan, 48109, United States.
[6]Macromolecular Science and Engineering Program, University of Michigan, Ann Arbor, Michigan, 48109, United States.
[7]Department of Orthopaedic Surgery, University of Michigan, Ann Arbor, Michigan, 48109, United States.
[8]Department of Mechanical and Materials Engineering, University of Alabama at Birmingham, Birmingham, Alabama 35294
[9]Department of Orthopaedic Surgery, Heersink School of Medicine, University of Alabama at Birmingham, Birmingham, Alabama 35294

[*] Corresponding author

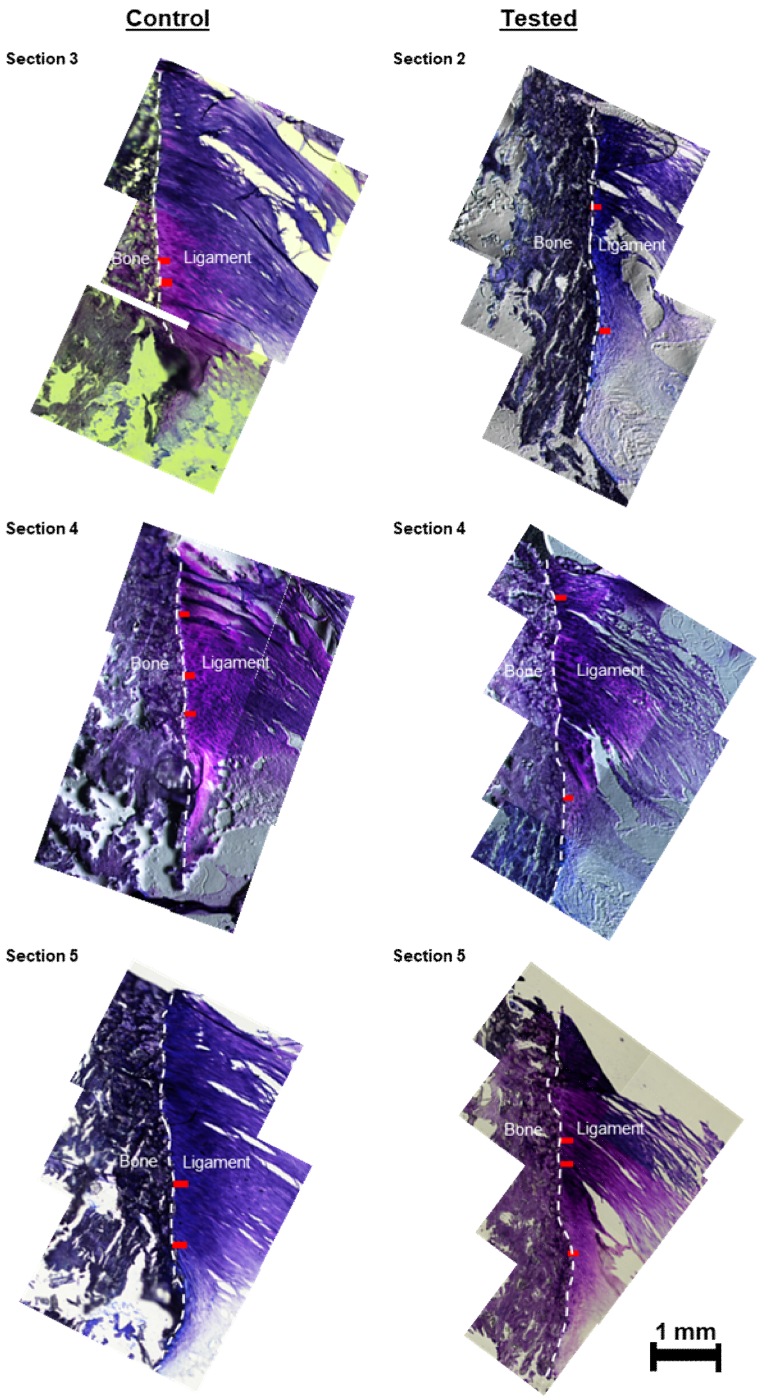

Supplementary Fig. 1. Toluidine blue stained ACL sections from cadaver sample I viewed under Brightfield Microscope 5x magnification. The bone and ligament regions are identified on the images with the ligament-to-bone insertion interface (tidemark) highlighted by the dashed white line. AFM-IR images are then acquired at multiple locations from the tidemark to the ligament as identified by the red rectangles in the ACL sections.

Supplementary Table 1. Summary Information for all cadaver samples

Samples	Gender	Age	Type	Number of sections	Number of ROI	Characteristic of ROI	Frequency peaks fitted	Lowest peak	Highest peak
I	Female	42	Control	3	7	5 x 50 μm ROI obtained using adjacent 5 x 25 μm and 5 x 26 μm windows with 1 μm overlap for image stitching	1, 2, 3, 4, 5, 6, 7	1	7
			Tested	3	7		1, 2, 3, 4, 5, 6, 7	1	7
II	Male	25	Control	3	4	5 x 5 μm ROI at 40 μm and 80 μm interval from tidemark	2, 3, 4, 5, 6, 7	2	7
			Tested	2	4		1, 2, 3, 4, 5, 6, 7	1	7
III		32	Control	3	3		2, 3, 4, 5, 6	2	6
			Tested	3	3		3, 4, 5, 6	3	6
IV		39	Control	4	4		2, 3, 4, 5, 6	2	6
			Tested	4	4		2, 3, 4, 5, 6, 7	2	7
V		47	Control	4	4		2, 3, 4, 5, 6, 7	2	7
			Tested	4	4		4, 5	4	-

Data from Sample I were collected with the most detailed imaging protocol and have significantly more data compared to all the data from Samples II, III, IV and V combined.

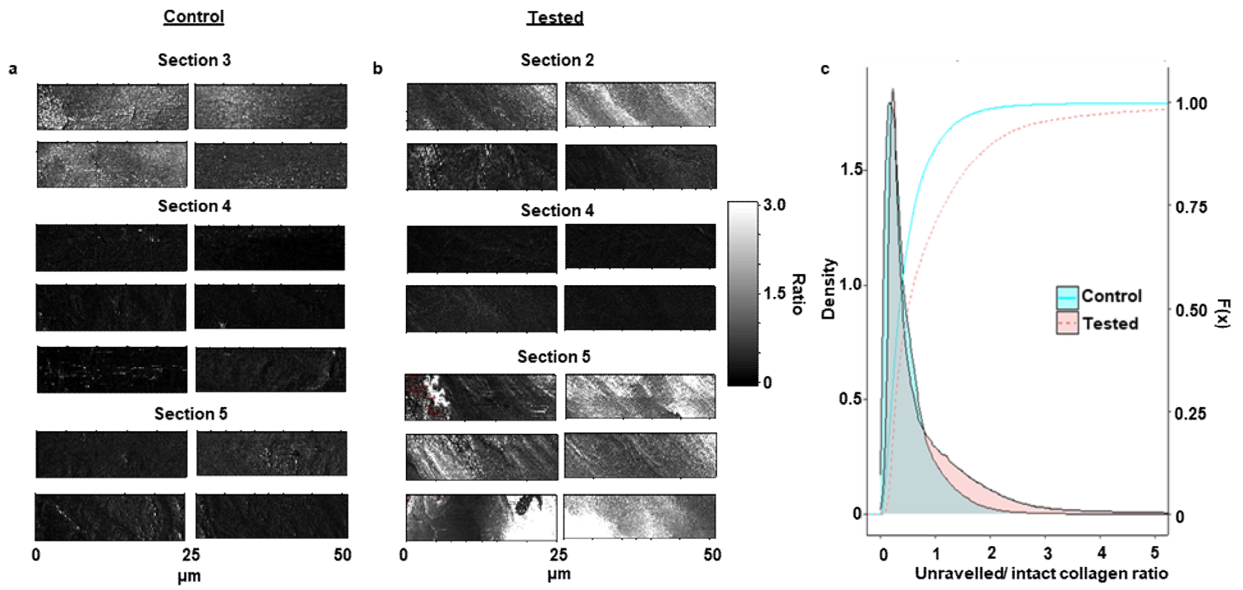

Supplementary Fig. 2. Overall analysis of Unravelled/ intact collagen ratio maps (1740/1680) comparing control and tested ACL from cadaver sample I. **a** Unravelled/ intact collagen ratio maps acquired from multiple tissue sections from the control ACL at different locations along the tidemark. **b** Unravelled/ intact collagen ratio maps acquired from the tested ACL from multiple tissue sections from the control ACL at different locations along the tidemark. Both ratio maps from control and tested show varying level of unravelled collagen between different tissues and locations along the tidemark. Varying morphologies of unravelled collagen domains are also observed in both control and tested ACL. **c** Quantitative comparison of kernel density distribution across all the control and tested ratio maps, with the solid blue and dashed red line representing the cumulative density function of all the control and tested ratio maps, respectively. Analysis shows a greater density of domains with high Unravelled/ intact collagen ratio in the tested ACL compared to the control ACL (KS test; $p < 2.2 \times 10^{-16}$). n = 2 human cadaver ACL.

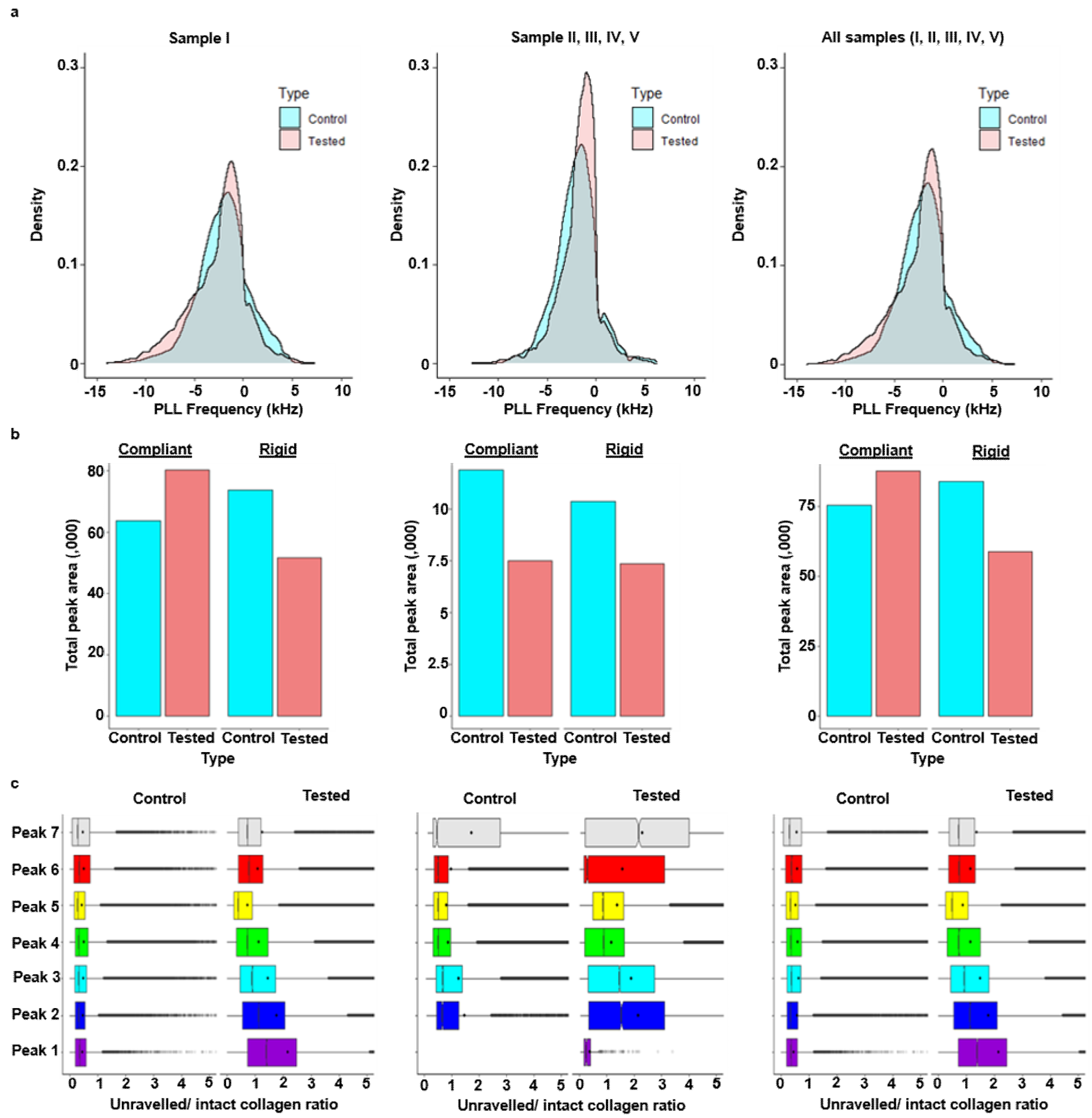

Supplementary Fig. 3 Comparison between cadaver sample I with cadaver samples II, III, IV and V, which were collected with a less detailed imaging protocol. **a** Overall kernel density distribution of all PLL frequency maps from cadaver sample I (left), sum of the cadaver samples II, III, IV and V (middle) and all cadaver samples I, II, III, IV and V (right). Analysis is divided into Sample I and Samples II-V because of the order of magnitude greater data for Sample I and different sampling protocol. **b** Quantifying the area of low frequency domain (< -5 kHz) and high frequency domain (> 0 kHz) in cadaver sample I, sum of the cadaver samples II, III, IV and V and all cadaver samples I, II, III, IV and V, which represent the compliant and rigid domains, respectively. Decrease in the most rigid domain as a result of mechanical testing holds up across both analysis of cadaver sample I (left) and the sum of the cadaver samples II, III, IV

and V (middle). Increase in compliant domain only holds up in the most extreme range of the tail; but not across bottom 15 kHz as seen in cadaver sample I. **c** Overall boxplot of unravelled/ intact collagen ratio in each of the fitted peaks/ stiffness levels across all the ACL sections of cadaver sample I (left), sum of the cadaver samples II, III, IV and V (middle) and all cadaver samples I, II, III, IV and V (right). Unravelled/ intact collagen ratio significant increases in tested ACL across cadaver sample I and the four other cadaver samples as measured by a one-tailed Mann-Whitney test ($p < 2.2 \times 10^{-16}$). n = 10 human cadaver ACLs.

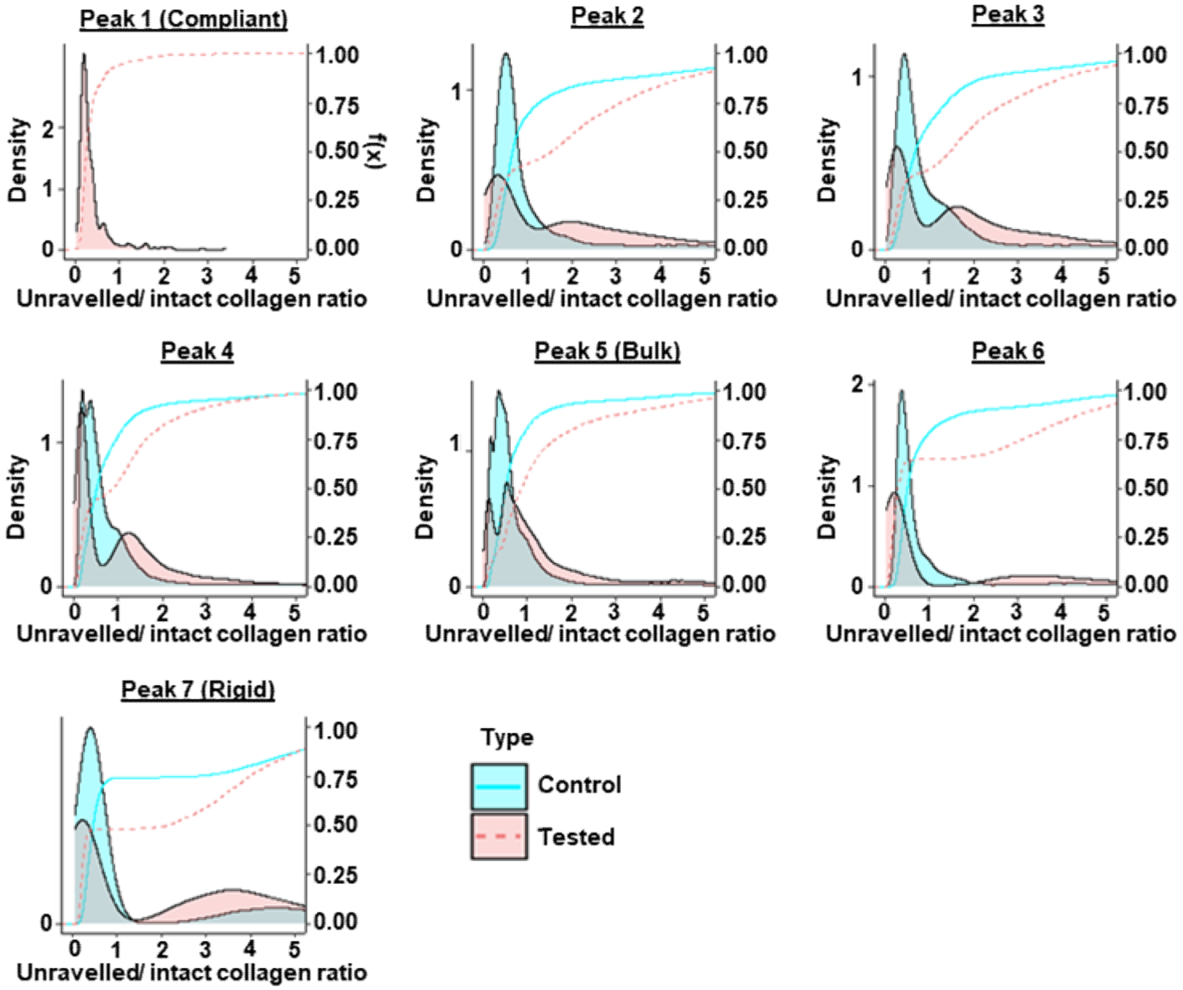

Supplementary Fig. 4 Overall kernel density distribution of unravelled/ intact collagen ratio across all the ratio maps from cadaver samples II, III, IV and V as a function of fitted peaks. Different peaks represent varying stiffness level with Peak 1 being the most compliant and Peak 7 being the most rigid. Greater density of high unravelled/ intact collagen ratio observed across all fitted peaks in the tested ACL compared to the control (KS test; $p < 2.2 \times 10^{-16}$). n = 8 human cadaver ACLs.

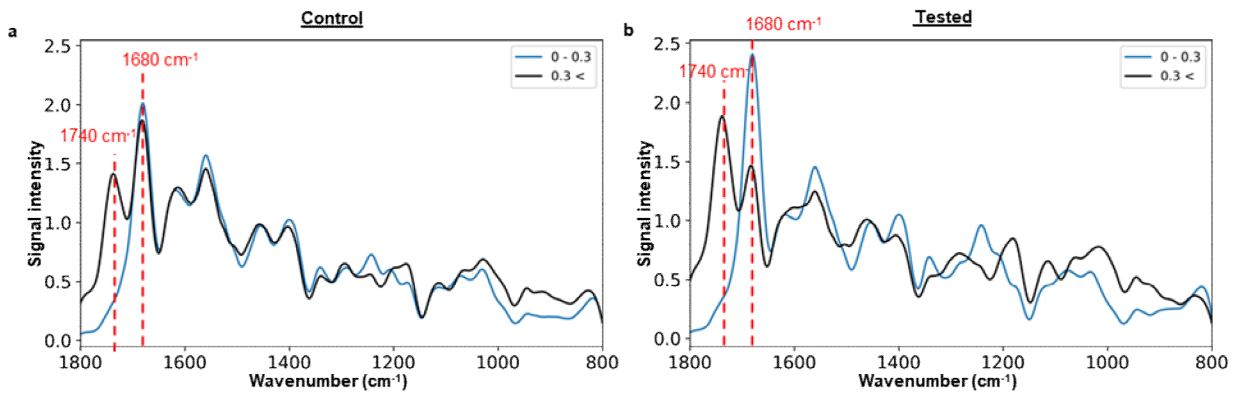

Supplementary Fig. 5 Comparison of average spectra with 1740/ 1680 ratio < 0.3 and 1740/ 1680 ratio > 0.3 across all cadaver samples in both control and tested ACL. a Averaging spectra with 1740/ 1680 ratio < 0.3 (blue) and 1740/ 1680 ratio > 0.3 (black) across all control cadaver samples. **b** Averaging spectra with 1740/ 1680 ratio < 0.3 (blue) and 1740/ 1680 ratio > 0.3 (black) across all tested cadaver samples. IR spectra with 1740/ 1680 ratio < 0.3 from both control and tested samples are consistent with typical shoulder at 1740 cm^{-1} in most IR data collected from collagenous tissue sample. n = 10 human cadaver ACLs.

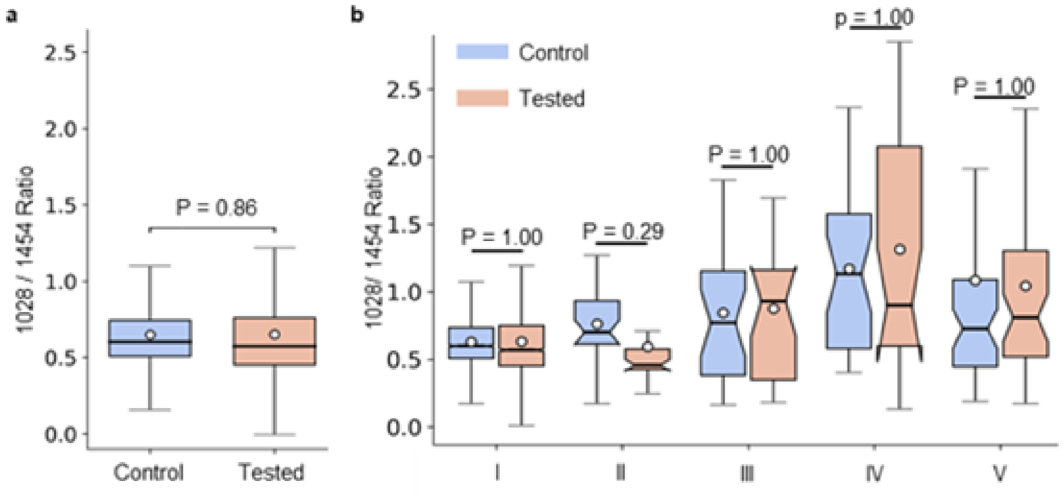

Supplementary Fig. 6 Comparison of normalization peak (1454 cm^{-1}) used to quantify changes in 1028 cm^{-1} (mineral) intensity. a Boxplot of 1028/1454 ratio between control and tested in all ACL samples which are not statistically different ($p = 0.86$). **b** Boxplot of 1028/1454 ratio between control and tested in individual ACL samples which are not statistically different between control and tested across each individual ACL samples. n = 10 human cadaver ACLs. (Box plot legend: dot = mean, solid line = median, whiskers = 5 – 95% of data, box = 1st – 3rd interquartile range, outliers not shown)

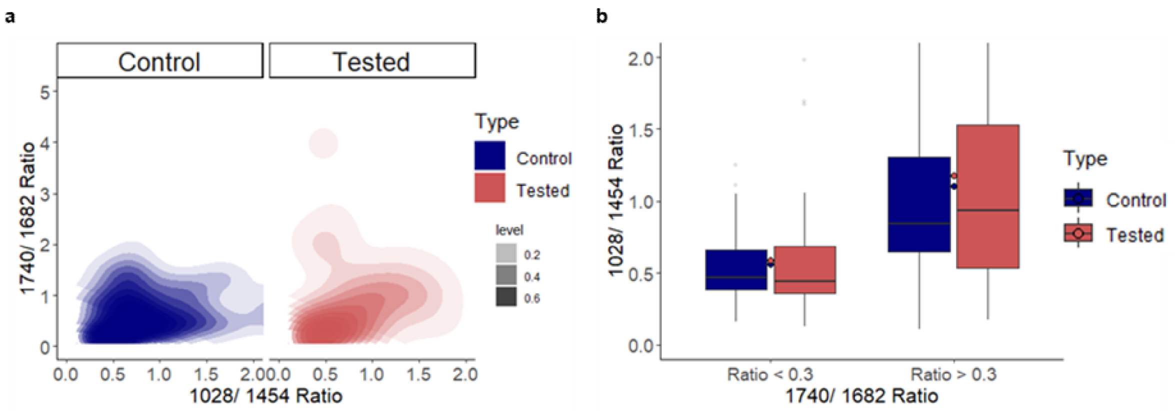

Supplementary Fig. 7 Overall analysis of mineral/ collagen CH_2 side chain ratio (1028/1454) and unravelled/ intact collagen ratio (1740/1682) comparing control and tested ACL across all the IR spectra from cadaver sample II, III, IV and V. **a** Comparison of overall 2D density plot of the mineral/ collagen CH_2 side chain ratio against unravelled/ intact collagen ratio between the control and tested ACL showing increasing collagen unravelling with increasing mineral ratio. **b** Boxplot of the mineral/ collagen CH_2 side chain ratio in domain with physiological level of collagen unravelling (ratio < 0.3) and domain with high level of collagen unravelling (ratio > 0.3) in both control and tested ACL. Domains with high level of collagen unravelling have significantly higher mineral ratio compared to domains with physiological level of collagen unravelling in both control and tested ACL (MWU; Control $p < 3.4 \times 10^{-6}$, Tested $p < 1.9 \times 10^{-7}$). n = 8 human cadaver ACLs.

Supplementary Fig. 8. Mechanical testing device for human cadaver knees.

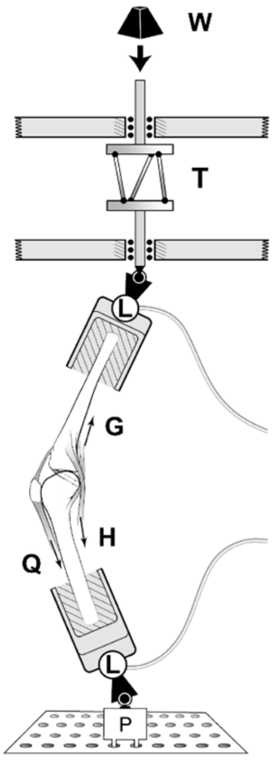

For full experimental details see:

Oh, Y. K., Kreinbrink, J. L., Wojtys, E. M., & Ashton-Miller, J. A. (2012). Effect of axial tibial torque direction on ACL relative strain and strain rate in an in vitro simulated pivot landing. Journal of Orthopaedic Research, 30(4), 528–534.

Oh, Y. K., Lipps, D. B., Ashton-Miller, J. A., & Wojtys, E. M. (2012). What strains the anterior cruciate ligament during a pivot landing? The American Journal of Sports Medicine, 40(3), 574–583.

Chen, J., Kim, J., Shao, W., Schlecht, S. H., Baek, S. Y., Jones, A. K., Ahn, T., Ashton-Miller, J. A., Banaszak Holl, M. M., & Wojtys, E. M. (2019). An anterior cruciate ligament failure mechanism. The American Journal of Sports Medicine, 47(9), 2067–2076.

Model Development for PLL Frequency fitting.

Carbon black/epoxy system

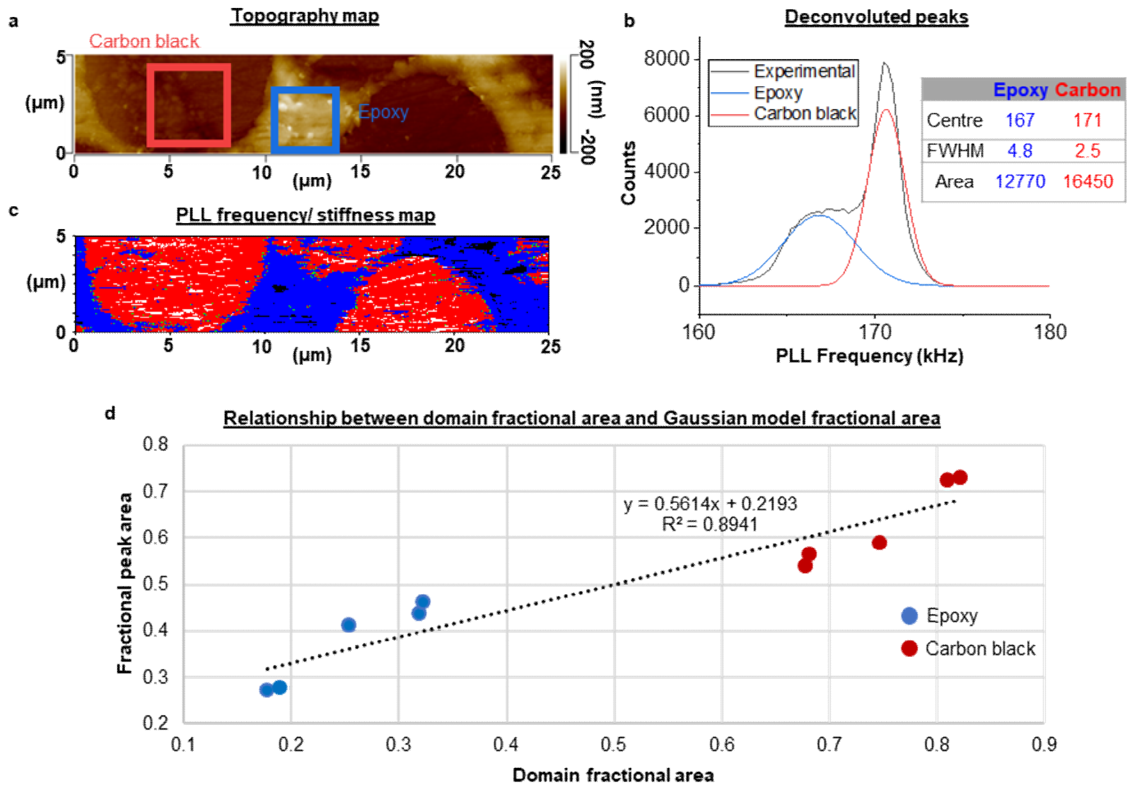

Supplementary Fig. 9 Validation of quantitative technique for stiffness analysis on carbon-epoxy standard sample. **a** 5 x 25 μm topography map collected using AFM-IR contact mode showing distinct carbon black domain (darker) and epoxy domain (lighter). **b** Applying peak fitting to deconvolute the multimodal distribution of PLL frequency map produced two peaks: the higher frequency peak (red) and the lower frequency peak (blue), representing the stiff carbon black and compliant epoxy, respectively. **c** A fitted PLL frequency map plotted using the peak center and Full Width Half Max (FWHM) of each peak to visualize the distinct stiffness domains. Identified stiffness domains are observed to align with the carbon black and epoxy domains identified through topography. **d** Deconvoluted peak area showed a strong correlation (R^2= 0.894) with the physical domain area in topography maps from five carbon-epoxy images.

ACL samples

a

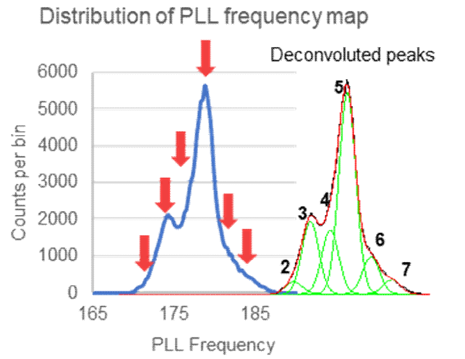

b

Peak index	Peak centre	FWHM	Peak area
2	172		847
3	174		5080
4	177	2.5	4488
5	179		14087
6	182		2593
7	184		991

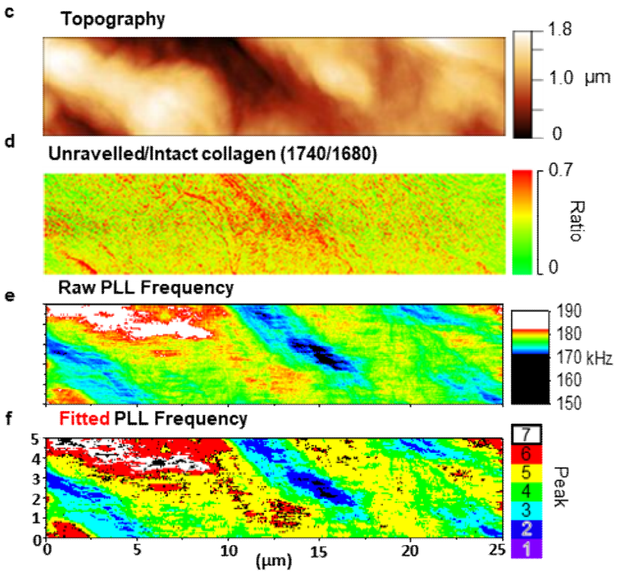

Supplementary Fig. 10 Example application of quantitative peak fitting technique on ACL sample. a PLL frequency distribution produced from the raw PLL frequency map (e) then deconvoluted with six peaks. **b** Statistical properties of fitted peak showing the center of each peak, full width half max (FWHM) and area under the peak. **c** Topography map. **d** Unraveled/ intact collagen ratio maps (1740/1680). **e** Raw PLL frequency map collected directly from instrument. **f** Fitted PLL frequency map generated from the peak fitting of the raw PLL frequency distribution (a) and (b). n = 1 human cadaver ACL.
